# Malvidin-3-*O*-Glucoside Ameliorates Cadmium-Mediated Cell Dysfunction in the Estradiol Generation of Human Granulosa Cells

**DOI:** 10.3390/nu15030753

**Published:** 2023-02-02

**Authors:** Shuer Liang, Xusheng Li, Ruijing Liu, Jun Hu, Yue Li, Jianxia Sun, Weibin Bai

**Affiliations:** 1 School of Chemical Engineering and Light Industry, Guangdong University of Technology, Guangzhou 510006, China; 2 Department of Food Science and Engineering, Institute of Food Safety and Nutrition, Jinan University, Guangzhou 510632, China; 3 The Sixth Affiliated Hospital, Jinan University, Dongguan 523576, China

**Keywords:** cadmium, anthocyanins, ovarian granular cells, KGN cells, malvidin-3-*O*-glucoside, estradiol

## Abstract

Cadmium (Cd) is a frequent environmental pollutant associated with biological toxicity that can harm female reproduction. Anthocyanins have been reported to reduce the toxicity of Cd. In the present study, the protective effects and underlying mechanisms of malvidin-3-*O*-glucoside (M3G) against the toxicity of Cd on female reproduction in KGN cells (human ovarian granulosa-like tumor cells) were investigated. After treating cells with 10 µmol/L cadmium chloride, the results showed that M3G lessened Cd-induced KGN cell cytotoxicity better than malvidin and malvidin-3,5-*O*-diglucoside. Additionally, M3G significantly decreased the Cd-induced generation of reactive oxygen species, inhibited the Cd-induced arrest of the G2/M phase of the cell cycle, and increased estradiol (E2) production. According to transcriptomic results, M3G reduced the abnormal expression of genes that responded to estrogen. Additionally, M3G promoted the endogenous synthesis and secretion of E2 by controlling the expression of CYP17A1 and HSD17B7. The current findings indicated that M3G is of great potential to prevent Cd-induced female reproductive impairment as a dietary supplement.

## 1. Introduction

Cadmium (Cd) is a toxic heavy metal that is widely used in manufacturing processes, such as batteries, pigments, and solder [1]. People are regularly exposed to Cd through smoking, industrial pollutants, and nutritional consumption [2,3]. As Cd accumulates in the body over time, it damages various tissues and organs and poses a serious health risk [4]. Exposure to Cd is intimately linked to the occurrence of several disorders, including female infertility [5]. Mounting evidence illustrated that women in Cd-contaminated areas experience irregular periods, difficult pregnancies, early deliveries, and stillbirths [6,7]. Additionally, animal studies also documented that Cd can threaten the reproductive system of female rats (0.09–4.5 mg/kg Cd orally per day) and mice (5–6 mg/kg Cd orally per day), manifesting as a broken ovary structure and disrupted estradiol (E2) secretion, which is responsible for the regular development and homeostasis of the female reproductive system [8,9,10]. It is worth noting that the ovarian granulosa cell, as the factory of estrogen production, is one of the targets of Cd in females, in which mitochondrial malfunction, estrogen attenuation, and induced apoptosis were persistent in the presence of Cd exposure [11].

Anthocyanins are present in a wide variety of fruits, vegetables, and flowers; they have been linked to significant health-promoting properties, such as anti-oxidant, anti-inflammation, anti-apoptosis, anti-cancer, and so on [12]. Researchers speculate that anthocyanins can directly chelate Cd under the intermolecular interaction at B-ring hydroxy group sites to lower Cd levels in mice and shield the body from the corresponding harm [13,14,15] Our previous study demonstrated that anthocyanins could ameliorate Cd-induced abnormal endometrial epithelial cell proliferation in mice by inhibiting estrogen response genes, thereby preventing Cd-induced female reproductive toxicity [10]. However, the effectiveness of anthocyanins in protecting against ovarian dysfunction induced by Cd is unclear. Ovarian granulosa cells are the somatic cells that surround the oocyte in the follicle and are required for follicular development and the synthesis of steroid hormones. Additionally, ovarian granulosa cells are linked to several ovarian disorders, including polycystic ovary syndrome and early ovarian failure [16]. Based on this, we conducted in vitro experiments with KGN cells (human ovarian granulosa-like tumor cells), to further investigate the bioactivity of anthocyanins against Cd-induced female reproductive toxicity and the corresponding mechanism. KGN cells can proliferate indefinitely in vitro and secrete small amounts of E2 [17]. Since the cell line has a similar steroid production mechanism to normal human granulosa cells, it is an excellent model for assessing female reproductive toxicity in vitro.

**Figure 1:**
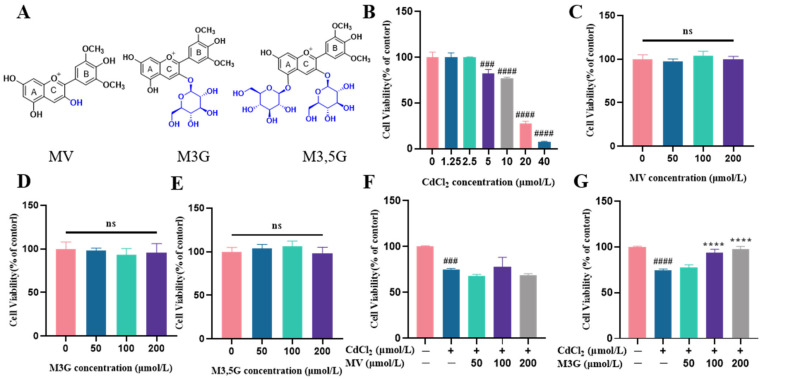

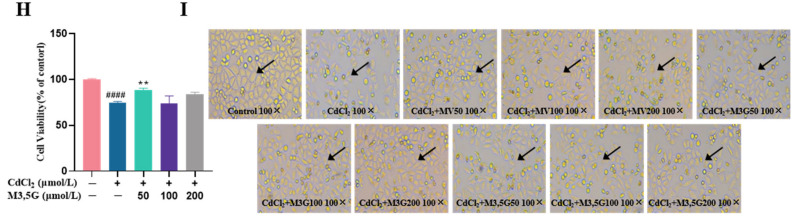
M3G increases CdCl_2_ exposure to KGN cell viability. (**A**) The structure of MV, M3G, and M3,5G. Cell viability of KGN cells incubated for 24 h with different compounds: (**B**) treated with CdCl_2_ (1.25–40 µmol/L); (**C**) treated with MV (50–200 µmol/L); (**D**) treated with M3G (50–200 µmol/L); (**E**) treated with M3,5G (50–200 µmol/L); (**F**) treated with 10 µmol/L CdCl_2_ and MV (50–200 µmol/L); (**G**) treated with 10 µmol/L CdCl_2_ and M3G (50–200 µmol/L); (**H**) treated with 10 µmol/L CdCl_2_ and M3,5G (50–200 µmol/L); and (**I**) cell morphology of KGN cells treated for 24 h with 10 µmol/L CdCl_2_ and 50–200 µmol/L MV, M3G, and M3,5G. Mean ± SD, *n* = 3. *^###^ p* < 0.001, *^####^ p* < 0.0001, vs. control; ** *p* < 0.01, **** *p* < 0.0001, vs. CdCl_2_-treated group. ns, no significant.

Since anthocyanins are rich in species in nature, in terms of the discrepant skeleton and glycoside substituents, the bioactivity of anthocyanins is completely different and structure-dependent. Malvidin-based anthocyanin is one of the most common anthocyanin families found in grapes and berries that have 3′,5′-dimethoxy substituents in the B-ring, which are very frequently accessible anthocyanins in the diet. Among them, malvidin (MV), malvidin-3-*O*-glucoside (M3G), and malvidin-3,5-*O*-diglucoside (M3,5G) are the most typical anthocyanins in this family (Figure 1A). Herein, we first compared the protective effect of three anthocyanins against Cd-induced KGN cell toxicology and investigated the possible underlying mechanisms of M3G considering the cell viability and estrogen secretion.

## 2. Materials and Methods

### 2.1. Cell Culture

KGN cells, human ovarian granulosa-like tumor cells, were purchased from the ATCC (Manassas, VA, USA); they were cultured on Dulbecco’s Modified Eagle Medium/Nutrient Mixture F-12 (DMEM/F12, Gibco, Rockville, MD, USA) containing 10% fetal bovine serum (Gibco, Rockville, MD, USA) and 1% penicillin/streptomycin/amphotericin (Gibco, Rockville, MD, USA) at 37 °C in 5% CO_2_ humidified chamber. 

### 2.2. Cell Viability

The stock solution of 200 µmol/L MV, M3G, and M3,5G (purity > 98%, ChemFaces, Wuhan, China) was prepared by dissolving the powder in DMSO. KGN cells were seeded at a density of 1 × 10^4^ cells per well in 96-well plates. The cells were exposed to different concentrations of MV, M3G, and M3,5G (50, 100, and 200 µmol/L) for 24 h after adhering for 12 h. The medium was discarded, and 100 µL of CCK8 solution (Beyotime Biotech, Shanghai, China) diluted 10 times with DMEM/F12 medium was added. The plates were then incubated at 37 °C for 2 h and measured using a microplate reader at 450 nm (Tecan, Mannedorf, Swiss). Cell viability tests were also used to investigate the effect of cadmium chloride (CdCl_2_) (1.25, 2.5, 5, 10, 20, and 40 µmol/L) on KGN cells, as well as the effects of MV, M3G, and M3,5G (50, 100, and 200 µmol/L) on 10 µmol/L CdCl_2_-treated KGN cells. Finally, the control group was used as the 100% cell viability; the value of other groups was calculated according to the measured absorption.

### 2.3. Cell Morphology Observation

KGN cells were seeded at a density of 1 × 10^4^ cells per well for 12 h in 96-well plates. Then 10 µmol/L CdCl_2_ was added, along with various concentrations of MV, M3G, and M3,5G (50, 100, and 200 µmol/L). Cell morphology was observed under an inverted phase microscope (Cnoptec, Chongqing, China) after 24 h of incubation.

### 2.4. Detection of E2

KGN cells were cultured at a density of 4 × 10^5^ cells per well in 6-well plates and were treated with 10 µmol/L CdCl_2_ and M3G (50, 100, and 200 µmol/L) for 24 h. The culture medium was collected after treatment and centrifuged for 10 min at 4 °C at 1000 rpm. The levels of E2 in the collected supernatant were determined using an E2 enzyme-linked immunosorbent assay (ELISA) kit (Cusabio, Wuhan, China) according to the manufacturer’s instructions.

### 2.5. Determination of Reactive Oxygen Species

The reactive oxygen species (ROS) levels in KGN cells were determined by a ROS assay kit (Beyotime Biotech, Shanghai, China). Cells were plated at a density of 1 × 10^4^ cells per well in 96-well plates, washed twice with phenol red-free and serum-free medium, and then incubated for 20 min at 37 °C with 30 µL of 2′,7′-dichlorofluorescein diacetate (DCFH-DA) solution diluted by the medium. After that, any extra DCFH-DA solution was washed away twice with phenol red-free and serum-free medium. Regarding the cells treated with 10 µmol/L CdCl_2_ and M3G (50, 100, and 200 µmol/L), the fluorescence of cells was assessed using a fluorescence plate reader (with emission and excitation wavelengths of 488 nm and 525 nm, respectively) at 10, 20, 40, 80, and 160 min.

### 2.6. Cell Cycle Analysis

For cell cycle measurements, KGN cells were plated in a 6-well plate at a density of 4 × 10^5^ cells per well for 12 h. After 24 h of treatment with 10 µmol/L CdCl_2_ and M3G (50, 100, and 200 µmol/L), trypsinization was used to harvest the cells, which were then fixed in 2 mL of 70% ice-cold ethanol overnight at −20 °C. The ethanol was entirely removed by centrifugation, and the cells were then washed with PBS. The PBS was then removed, and the cell cycle was examined using propidium iodide (PI), a fluorescent dye probe. For each sample, 8 × 10^3^ cells were collected and measured using a CytoFLEX flow cytometer (Beckman Coulter, Brea, CA, USA), which were then analyzed with ModFit software version 5.0 (Verity, Topsham, ME, USA).

### 2.7. Transcriptomic Analysis

Cells were seeded at 4 × 10^5^ per well in 6-well plates for 12 h; then, they were treated with 10 µmol/L CdCl_2_ and 200 µmol/L M3G separately or in combination for 24 h. After treatment, total RNA was extracted by the Trizol reagent (Beyotime Biotech, Shanghai, China) according to the manufacturer’s protocol. Then, RNA purity and integrity were assessed using the NanoDrop 2000 spectrophotometer (Thermo Scientific, Waltham, MA, USA) and the Agilent 2100 Bioanalyzer (Agilent Technologies, Santa Clara, CA, USA). Following the construction of the libraries with the TruSeq Stranded mRNA LT Sample Prep Kit (Illumina, San Diego, CA, USA), the library was sequenced on the Illumina HiSeq X Ten platform to generate a 150 bp paired-end read. Using Trimmomatic, raw reads in fastq format were processed to remove low-quality reads and obtain clean reads. Clean reads were mapped to the human genome (GRCh38) with HISAT2.

### 2.8. Quantitative Real-Time Polymerase Chain Reaction

KGN cells were seeded in a 6-well plate at a density of 4 × 10^5^ cells per well for 12 h and then treated with 10 µmol/L CdCl_2_ and 200 µmol/L M3G separately or in combination for 24 h. The extracted RNA was reverse-transcribed into cDNA with Evo M-MLV Reverse Transcription Kit (Accurate Biology, Hunan, China), followed by RT-PCR using SYBR Green Premix Pro Taq HS qPCR Kit (Accurate Biology, Hunan, China). Based on NCBI Primer-Blast, primers were designed for RT-qPCR and are listed in Table 1. Then, the QuantStudio real-time PCR system (Thermo Fisher, Waltham, MA, USA) was used for PCR amplification and the response procedures were as follows: 95 °C for 30 s, 40 cycles of 95 °C for 5 s, and 60 °C for 30 s. Relative target gene mRNA expression was analyzed by the 2^−ΔΔCt^ method.

### 2.9. Western Blot

The cells were cultured in 6-well plates at a density of 4 × 10^5^ cells per well and exposed for 24 h to 10 µmol/L of CdCl_2_ and 200 µmol/L of M3G. Following this, the KGN cells were lysed in radioimmunoprecipitation assay (RIPA) buffer containing phenylmethanesulfonyl fluoride (PMSF) and protease inhibitors and were centrifuged for 10 min at 12,000 rpm. The BCA method (Applygen, Beijing, China) was used to determine the protein concentration of the samples. Then, using PBS and protein loading buffer, 2.5 μg/μL of protein samples for separation were prepared. For each sample, 20 µg of protein were subjected to 10% SDS-PAGE and then transferred to polyvinylidene difluoride (PVDF) membranes. Primary antibodies HSD3B (Abcam, Cambridge, MA, USA), CYP17A1 (Abclonal, Wuhan, China), HSD17B7 (Proteintech, Wuhan, China) at 1:1000 dilution, and GAPDH (Proteintech, Wuhan, China) at 1:5000 dilution were incubated overnight at 4 °C with blots, followed by incubation with the horseradish peroxidase (HRP)-conjugated secondary antibodies (Proteintech, Wuhan, China). The protein bands were detected using an imaging analysis system (Tanon, Shanghai, China) and quantified using Image J software (BioTechniques, NY, USA).

### 2.10. Statistical Analysis

The data are expressed as the mean ± standard deviation (SD). GraphPad Prism 8 software conducted the differences between groups through a one-way ANOVA. Results with a *p* < 0.05 indicate statistical significance. Each experiment included three replications.

## 3. Results

### 3.1. Effects of M3G on the Viability and Morphological Changes of CdCl_2_-Exposed KGN Cells

The impact of various CdCl_2_ concentrations on cell viability in KGN cells was investigated. The CCK8 assay revealed that CdCl_2_ concentrations of 1.25 and 2.5 µmol/L did not have a significant impact on cell viability. In the subsequent concentrations, CdCl_2_ decreased cell viability in a dose-dependent manner, with cell viability decreasing to 82.5% for 5 µmol/L and 76.9% for 10 µmol/L, respectively. At the 20 and 40 µmol/L doses, CdCl_2_ toxicity was observed at 27.4% and 7.8% (Figure 1B), indicating that Cd causes severe damage to KGN cells. Altogether, 10 µmol/L of CdCl_2_ was used to treat the KGN cells in the following experiments. 

There was no significant change in cell viability following a 24 h exposure to MV, M3G, or M3,5G (Figure 1C–E). Furthermore, it was discovered that MV treatment at any concentration did not lessen CdCl_2_-induced cytotoxicity (Figure 1F). However, M3G at 100 and 200 µmol/L, and M3,5G at 50 µmol/L, showed significant protective effects, with cell viability values increased to 94.2%, 97.7%, and 88.8%, respectively (Figure 1G,H). Consequently, 50, 100, and 200 µmol/L M3G were considered for the remaining measurements due to the best protective effects.

Using an inverted phase microscope, the cell morphology of KGN cells that had been exposed to 10 µmol/L CdCl_2_ and different concentrations of MV, M3G, and M3,5G (50, 100, and 200 µmol/L) was observed. The cells in the control group grew more densely and with an irregular polygon morphology, as seen in Figure 1I. The cells became small and round after 24 h of CdCl_2_ treatment and cell adhesion deteriorated, resulting in a gradual decrease of adherent cells and an increase of floating cells. The M3G treatment group (treatment with 10 µmol/L CdCl_2_ and M3G at various concentrations) improved cell morphology and cell adhesion compared with the CdCl_2_ treatment. Moreover, there was no observable difference in cell growth morphology and distribution between the MV, M3,5G treatment group (treatment with 10 µmol/L CdCl_2_ and MV, M3,5G at various concentrations) and CdCl_2_ treatment groups when considering the cell size, density, and shape.

### 3.2. Effects of M3G on E2 Levels of CdCl_2_-Exposed KGN Cells

**Figure 2:**
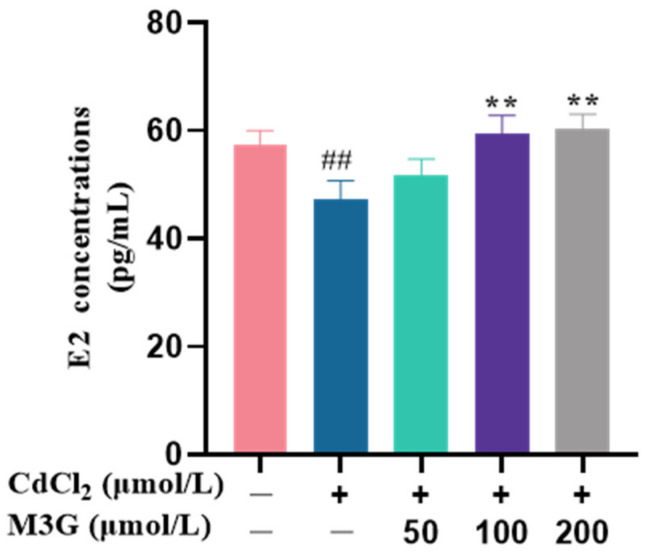
M3G increases E2 levels in CdCl_2_-exposed KGN cells. E2 concentrations of KGN cells treated with 10 µmol/L CdCl_2_ and 50-200 µmol/L M3G for 24 h. Mean ± SD, *n* = 3. *^##^ p* < 0.01, vs. control; ** *p* < 0.01, vs. CdCl_2_-treated group.

Since Cd treatment is detrimental to the cell survival quality, we further examined the function of the reserved cells, regarding that E2 synthesis is the critical biofunctionability of KGN cells. Therefore, the cells were subjected to 10 µmol/L of CdCl_2_, and M3G as the efficient anthocyanin was considered to ameliorate the E2 production in vitro. As shown in Figure 2, the E2 level in the control group was 57.4 ± 2.6 pg/mL, which was reduced to 47.2 ± 3.6 pg/mL following CdCl_2_ treatment and was significantly different. The E2 levels were noticeably higher, at 59.6 ± 3.3 pg/mL and 60.5 ± 2.6 pg/mL, when the M3G dose was 100 µmol/L and 200 µmol/L, respectively. This indicated that M3G protected E2 production in KGN cells exposed to CdCl_2_.

### 3.3. Protective Effect of M3G against ROS Production Induced by CdCl_2_

**Figure 3:**
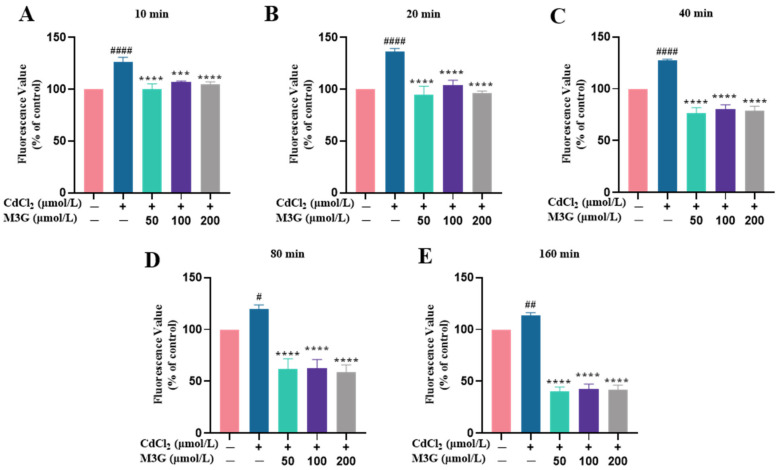
M3G reduces ROS levels in CdCl_2_-exposed KGN cells. ROS levels of KGN cells were treated for (**A**) 10, (**B**) 20, (**C**) 40, (**D**) 80, and (**E**) 160 min with 10 µmol/L CdCl_2_ alone and in combination with 50-200 µmol/L M3G. Mean ± SD, *n* = 3. *^#^ p* < 0.05, *^##^ p* < 0.01, *^####^ p* < 0.0001, vs. control; *** *p* < 0.001, **** *p* < 0.0001, vs. CdCl_2_-treated group.

ROS are present in cells and are essential in several cellular signaling pathways. However, excess ROS can result in oxidative stress, which ultimately impairs cellular function [18]. During the present experiment, DCFH-DA fluorescent dye was used to detect ROS produced by treated KGN cells with 10 µmol/L CdCl_2_ and 50–200 µmol/L M3G. The intensity of the fluorescence rose with the number of ROS produced by cell generation. In the CdCl_2_-treated group, the ROS production of KGN cells was increased significantly, with a fluorescence intensity of 126.5% at 10 min, and peaked at 20 min with an intensity of 136.5%. ROS production by cells in the CdCl_2_ group was decreased after 40 min, and their fluorescence intensity was reduced to 127.3%, 120.0%, and 113.3%, at 40 min, 80 min, and 160 min, respectively (Figure 3). However, ROS production in KGN cells was significantly reduced after it interfered with M3G at concentrations of 50–200 µmol/L, indicating that M3G scavenged excess ROS from CdCl_2_-exposed KGN cells. The fluorescence intensities of M3G at 50, 100, and 200 µmol/L concentrations were 100.0%, 107.0%, and 104.6% at 10 min, and decreased to 95.0%, 104.0%, and 96.6% at 20 min, and 76.7%, 80.4%, and 78.6% at 40 min, respectively. This result showed that 50 µmol/L of M3G had the best ability to scavenge ROS during the first 40 min. In contrast, M3G at 50 and 100 µmol/L concentrations exhibited fluorescence intensities of 61.9% and 62.5%, respectively, while at 80 min; M3G at 200 µmol/L concentration had the strongest ability to scavenge ROS with a fluorescence intensity of 59.0%. The M3G-treated group cell fluorescence intensity reached a level of about 41% by 160 min.

### 3.4. Protective Effect of M3G on Cell Cycle Arrest of CdCl_2_-Exposed KGN Cells

**Figure 4:**
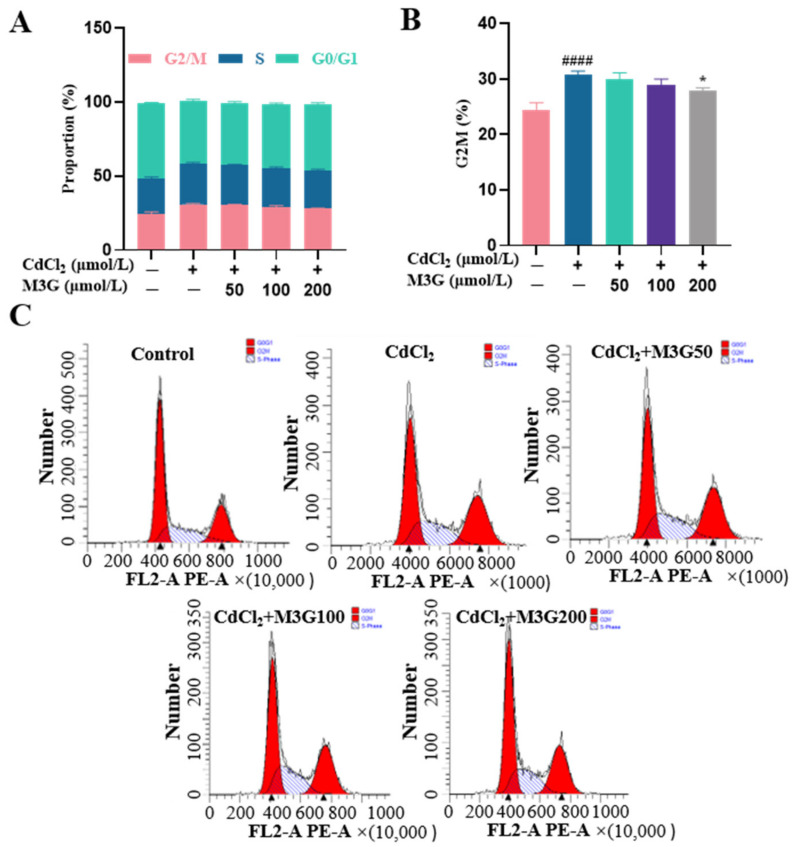
M3G inhibits the G2/M phase arrest caused by CdCl_2_ exposure to KGN cells. Cell cycle of KGN cells after 24 h of exposure to 10 µmol/L CdCl_2_ and 50-200 µmol/L M3G; (**A**) analysis of the cell cycle phases, the bar graph showing the G2/M, S, and G0/G1 phases; (**B**) analysis of the cell cycle phases, the bar graph showing the percentage of cells in G2/M phases; and (**C**) analysis of the cell cycle distribution. Mean ± SD, *n* = 3. *^####^ p* < 0.0001, vs. control; * *p* < 0.05, vs. CdCl_2_-treated group.

The cell cycle, which includes the G0 (quiescent phase), G1 (early DNA synthesis phase), S (DNA synthesis phase), G2 (late DNA synthesis phase), and M phase (division phase), is the stage sequence of cell proliferation. Evidence suggests that Cd damages cellular DNA, causing cells to halt their cell cycle at the G1-S or the G2-M transition point [19]. The cells never undergo mitosis if the damage cannot be repaired; instead, they enter transiently quiescence, senescence, or even death. As shown in Figure 4, flow cytometry analysis revealed that after 24 h of incubation with CdCl_2_, KGN cells exhibited a significant increase in cell percentages in the G2/M phase, indicating that CdCl_2_ arrested the cell cycle. M3G inhibited cell cycle G2/M phase arrest after 24 h treatment with 10 µmol/L CdCl_2_ and 200 µmol/L M3G.

### 3.5. Effects of M3G on the Transcriptome of CdCl_2_-Exposed KGN Cells

**Figure 5:**
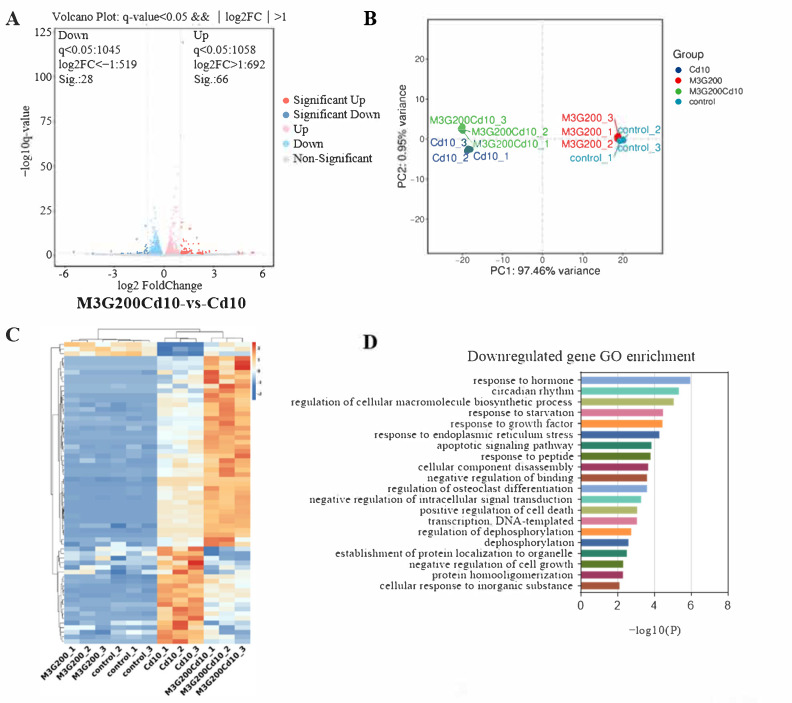

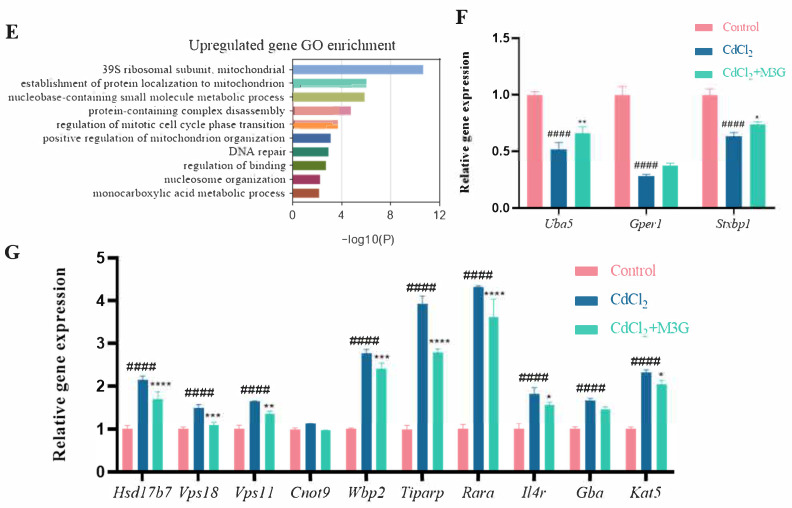
Transcriptome after CdCl_2_ treatment alone and in combination with M3G. RNA sequencing and bioinformatics analysis of KGN cells following treatment with 10 µmol/L CdCl_2_ and 200 µmol/L M3G separately or in combination: (**A**) differential gene volcanic maps; (**B**) the PCA analysis results; (**C**) hierarchical clustering heat map of differentially expressed genes; (**D,E**) GO classification of differentially expressed enrichment; Estrogen-sensitive genes selected from the RNA sequencing analysis; (**F**) the upregulated gene expression after CdCl_2_ + M3G treatment; and (**G**) the downregulated gene expression after CdCl_2_ + M3G treatment. Mean ± SD, *n* = 3. *^####^ p* < 0.0001, vs. control; * *p* < 0.05, ** *p* < 0.01, *** *p* < 0.001, **** *p* < 0.0001, vs. CdCl_2_-treated group.

**Figure 6:**
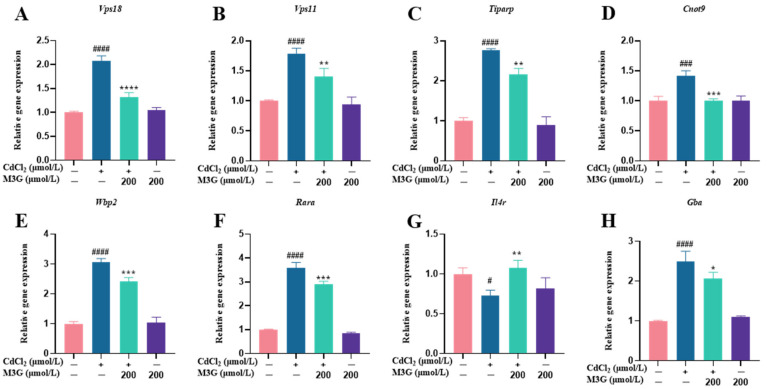

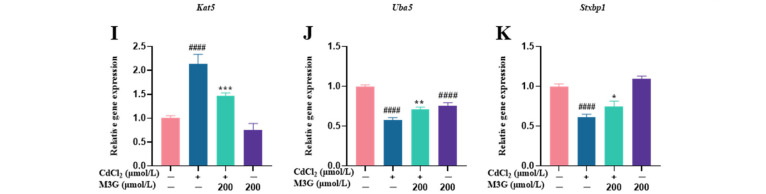
M3G ameliorated the abnormal expression of estrogen-sensitive genes caused by CdCl_2_ exposure to KGN cells. Gene expression of KGN cells after treatment with 10 µmol/L CdCl_2_ and 200 µmol/L M3G alone or in combination. Gene expression of (**A**) *Vps18*, (**B**) *Vps11*, (**C**) *Tiparp*, (**D**) *Cnot9*, (**E**) *Wbp2*, (**F**) *Rara*, (**G**) *Il4r*, (**H**) *Gba*, (**I**) *Kat5*, (**J**) *Uba5*, and (**K**) *Stxbp1*. Mean ± SD, *n* = 3. *^#^ p* < 0.05, *^###^ p* < 0.001, *^####^ p* < 0.0001, vs. control; * *p* < 0.05, ** *p* < 0.01, *** *p* < 0.001, **** *p* <0.0001, vs. CdCl_2_-treated group.

KGN cells were subjected to transcriptomic analysis to identify potential mechanisms causing changes in response to CdCl_2_ exposure (10 µmol/L) and the protective effect of M3G (200 µmol/L). As shown in the volcano map in Figure 5A, the combined CdCl_2_ and M3G treatment group altered a few genes; 94 genes had a differential expression, with 66 upregulated and 28 downregulated. Analysis of gene ontology (GO) enrichment pathways revealed a variety of enrichment biochemical pathways linked to differentially expressed genes, in the CdCl_2_-only group and the CdCl_2_-M3G group, including pathways in response to hormones, apoptotic signaling pathways, response to endoplasmic reticulum stress, and positive regulation of mitochondrial organization (Figure 5D,E). Based on the abnormal secretion of E2 by KGN cells due to Cd and our interest, 13 estrogen-sensitive genes were selected from the RNA-seq data and further validated by RT-qPCR analysis (Figure 5F,G). As compared to CdCl_2_-only treatments, CdCl_2_-M3G-treated groups showed decreased expressions of ten genes and increased expressions of three genes (Figure 6). Among them, the gene expression of *Gper1* exhibited a trend of significant decrease after Cd treatment, but there was no significant difference after M3G treatment. The expression of *Hsd17b7*, a key gene in the estradiol synthesis pathway, showed a significant upward trend after Cd treatment and decreased significantly after the co-treatment of Cd and M3G. Meanwhile, the gene expression trends of *Rara* and *Wbp2* were the same as *Hsd17b7*, and their transcriptomic data and qPCR results were identical. However, although the transcriptomics and qPCR data of *Cnot9* showed the same trend of increasing after Cd treatment and then decreasing after the addition of M3G co-treatment, none of the transcriptomic trend changes were significantly different. The results supported our hypothesis that M3G may protect the KGN cells from the cytotoxic effects of Cd exposure by inhibiting the effects of Cd on the expression of these E2-responsive genes and demonstrated that the trends in gene expression were consistent with RNA-Seq results.

### 3.6. Effects of M3G on E2-Related Protein and Gene Expression of CdCl_2_-Exposed KGN Cells

**Figure 7:**
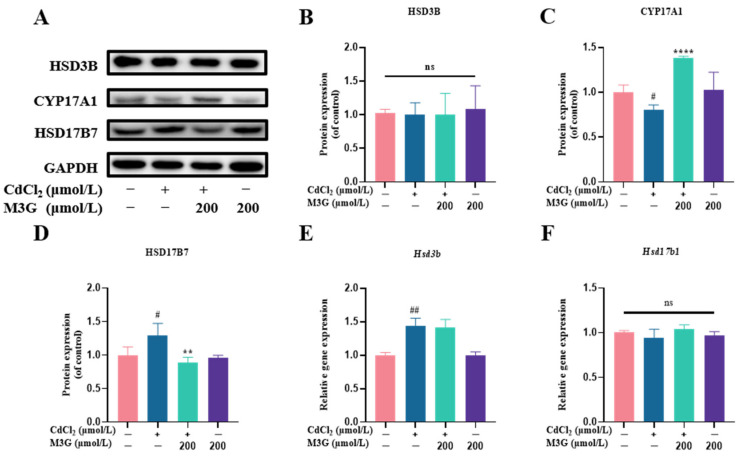

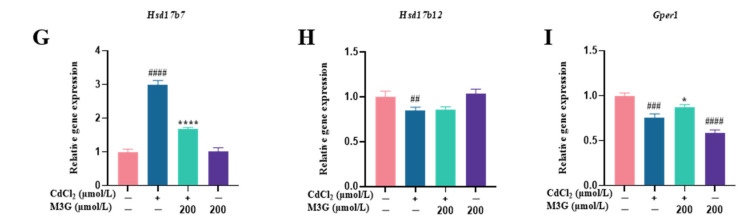
M3G ameliorated the abnormal expression of CYP17A1 and HSD17B7 caused by CdCl_2_ exposure to KGN cells. Protein and gene expression of KGN cells after treatment with 10 µmol/L CdCl_2_ and 200 µmol/L M3G alone or in combination: (**A**) protein expression of HSD3B, CYP17A1, and HSD17B7; quantification of band intensity of (**B**) HSD3B, (**C**) CYP17A1, and (**D**) HSD17B7 normalized to GAPDH; gene expression of (**E**) *Hsd3b*, (**F**) *Hsd17b1*, (**G**) *Hsd17b7*, (**H**) *Hsd17b12*, and (**I**) *Gper1*. Mean ± SD, *n* = 3. *^#^ p* < 0.05, *^##^ p* < 0.01, *^###^ p* < 0.001, *^####^ p* < 0.0001, vs. control; * *p* < 0.05, ** *p* < 0.01, **** *p* <0.0001, vs. CdCl_2_-treated group. ns, no significant.

Western blot (WB) and RT-qPCR techniques were used to measure the expression levels of critical proteins and genes in E2 synthesis in KGN cells to learn more about the potential mechanism of abnormal E2 secretion caused by Cd. As shown in Figure 7B,C, the expression of HSD3B protein was unchanged under any treatment, whereas 200 µmol/L M3G reversed the trend of decreased CYP17A1 protein expression in KGN cells due to CdCl_2_ exposure. Furthermore, CdCl_2_ increased HSD17B7 expression in KGN cells, while M3G at 200 µmol/L decreased it (Figure 7D). In terms of gene expression levels, gene expression was significantly higher for *Hsd3b*, comparable across groups for *Hsd17b1*, significantly lower for *Hsd17b12*, consistent for *Hsd17b7* for both gene and protein expression, and lowered, followed by an increasing trend for *Gper1* (Figure 7E–I).

## 4. Discussion

Cd is a widely occurring metal and environmental contaminant that harms the female reproductive system in various ways. Particularly, serving as the factory of estrogen production, the human ovarian granular cell has been well identified as the target of Cd toxicity [11]. Anthocyanins have recently attracted substantial attention as potent nutrients for the treatment or prevention of reproductive system harm brought on by Cd [10]. Since malvidin is the primary anthocyanin that is abundant in dietary fruits and vegetables [20], malvidin-based anthocyanins with different glycosides were considered in the present study. This study aimed to compare the protective effect of MV, M3G, and M3,5G against Cd-induced cell toxicity in KGN cells; as well as investigate whether M3G could lessen the impact of Cd on the production of E2 and the potential mechanism.

An in vitro model of KGN cell injury induced by 10 µmol/L CdCl_2_ was established and the protective effects of various molecular structural anthocyanins, including MV (C3-OH on a C-ring), M3G (C3-O-glc on a C-ring), and M3,5G (C3-O-glc on an A-ring and C5-O-glc on a C-ring) were evaluated. The structure of anthocyanins has a substantial impact on the suppression of aberrant proliferation of injured cells. Previous studies have shown that in a damaged cell model, anthocyanins with the monosaccharose substitution dissolved in DMSO had a significantly stronger protective effect than those with the disaccharide substitution against oxLDL-induced endothelial injury associated with increased cell viability, promoted NO release, and so on [21]. Analogously, C3 position glycosylation on anthocyanin dramatically increased its inhibitory effect on tumor cells [22]. Consistent with these observations, M3G was more effective than MV and M3,5G in reducing cytotoxicity when applied to KGN cells exposed to Cd, indicating that the monosaccharose structure has a stronger protective effect against Cd damage.

Cd exposure was found to significantly enhance oxidative stress in ovarian granulosa cells, playing a causal role in Cd-induced reproductive toxicity [11,23]. Therefore, reducing oxidative stress was considered an ideal therapy to partially intercept reproductive system damage. The results of the current study showed that M3G has the propensity to balance Cd-induced oxidative stress, represented as an immediate and significant reduction in ROS generation. More concretely, the reduced ROS formation could be attributed to the phenolic hydroxyl groups on the anthocyanin ring that can scavenge the highly reactive state of free radicals, thereby lessening or eliminating oxidative stress conditions [24,25].

Cell proliferation requires an orderly cell cycle progression. In the present study, we investigated the cell cycle changes induced by Cd. After 24 h of 10 µmol/L CdCl_2_ treatment, the results revealed an increased number of cells in the G2/M phase. Given that Cd is recognized as a spindle poison, Cd can stop mitosis and is more sensitive to attack cells in the G2/M phase [26,27]. However, once Cd accumulates in cells and a G2/M phase arrest is formed; the arrest state is irreversible regardless of whether Cd is taken out of the culture medium or continuously supplied [28]. Additionally, it has been demonstrated that oxidative damage also contributes to cell cycle arrest and represses cell proliferation [29,30]. Anthocyanins have been shown to alleviate G2/M phase arrest in a variety of cellular injury systems because they prevented cell cycle perturbation caused by cytotoxicity and produced a normal cell cycle pattern [31,32]. Similarly, 200 µmol/L M3G treatment prevented G2/M phase arrest in the present experiments, protecting KGN cells from Cd-induced damage.

Granulosa cells are responsible for estrogen (steroid hormones) synthesis and secretion in females. The three primary endogenous forms of estrogen are estrone (E1), estradiol (E2), and estriol (E3), which are in charge of the development and regulation of the female reproductive system and the maintenance of its function. Particularly E2 is the master and most biologically active estrogen [33]. Earlier research has demonstrated that Cd exposure is significantly linked to ovarian dysfunction by interfering with E2 secretion [34]. In agreement with this finding, we discovered that the E2 levels in the Cd-exposed group were significantly lower than those in the control group; while M3G therapy appeared to reverse the aberrant hormone release in KGN cells as evidenced by the much greater E2 content in the CdCl_2_ + M3G group.

In light of the improved E2 level after M3G intervention, the biogenesis of steroids involving a variety of steroidogenic enzymes was subsequently investigated. The first step in steroidogenesis is the transformation of cholesterol into pregnenolone, which is then changed into progesterone by HSD3B (3β-hydroxysteroid dehydrogenase). With the assistance of CYP17A (17α-hydroxysteroid dehydrogenase), progesterone is further transformed into androgens, such as androstenedione, testosterone, and dehydroepiandrosterone. In addition, androgens are converted to estrogen in granulosa cells, where androstenedione is converted to E1, which is then converted to E2 by the enzyme HSD17Bs (17β-hydroxysteroid dehydrogenase). Changes in the expression of steroidogenic enzymes influence steroid hormone production [35]. HSD3B and CYP17A1 expression are crucial for the production of estrogen because progesterone and androgen are precursors to the hormone. Previous research has found that Cd exposure decreases E2 secretion from human granulosa cells, as well as HSD3B gene and protein expression levels [36]. Another study has demonstrated that Cd harms Leydig cells and reduces CYP17A1 expression [37]. Contrary to these findings, the HSD3B gene expression in the present study was increased, while protein expression did not differ significantly (Figure 7B). Moreover, the direct effects of Cd on post-transcriptional and translational processes, as well as enzymatic activities, may also be responsible for variations in the gene and protein expression of E2 secretion and HSD3B.

The conversion of E1 to E2 is catalyzed by the HSD17Bs’ enzymes family including HSD17B1, HSD17B7, and HSD17B12 [38,39]. HSD17B1 is the most active enzyme during E2 production, however, the expression of HSD17B1 in this study did not significantly differ from controls, indicating that Cd did not affect E2 synthesis by changing HSD17B1 expression. Additionally, the HSD17B12 enzyme is crucial for ovarian health and its decreased expression can result in early ovarian failure and ovulation problems. However, it has also been shown that decreased HSD17B12 expression has no impact on ovarian E2 synthesis [38]. Surprisingly, the transcriptomic data showed that the expression of HSD17B7 showed a trend of increasing after Cd exposure and decreasing after M3G treatment; the results of RT-qPCR and WB experiments supported this trend as well. In conjunction with the E2 levels, it was also demonstrated that HSD17B7 regulates estradiol secretion via negative feedback mechanisms. As a result, M3G could alleviate the abnormal estradiol synthesis and secretion brought on by Cd by enhancing the expression of CYP17A1 and HSD17B7 proteins in KGN cells.

Estrogen receptors are necessary for reproduction, and their absence not only disrupts hormone signaling but also affects follicular growth. The estrogen receptors include estrogen receptor alpha (ERα), estrogen receptor beta (ERβ), and G-protein coupled estrogen receptor 1 (GPER1) [40]. GPER1, also known as GPR30, is a recognized membrane estrogen receptor that is expressed in the uterus and ovaries; it can bind with high affinity, and signal in response to E2 [41]. Retinoic acid receptor alpha (RARA) is a crucial element of the estrogen receptor (ER) transcriptional complex, which controls the expression of estrogen target genes [42]. WW domain binding protein 2 (WBP2) is thought to be an activator of ER and enhances the trans-activation of the ER [43]. In this study, Cd caused a decrease in GPER1 expression, an effect that was avoided when M3G was added. For RARA and WBP2, M3G reduced the Cd-induced high expression. However, it appears that the underlying mechanisms of action of E2 and GPER1, RARA, and WBP2 are complicated, and further investigation is necessary to elucidate them. 

Together, the present result demonstrated the critical role of M3G in the protection of Cd-induced dysfunction of granule cells. Considering the application of anthocyanins in dietary supplementation, pure M3G is expensive and hardly accessible, and the anthocyanins extracts containing M3G, or other types of functional anthocyanins targeted to granule cells, could be considered after the in vitro and in vivo verification. More studies related to the improvement of M3G accessibility to ovary granule cells and the corresponding animal study should be conducted in the future.

## 5. Conclusions

In conclusion, the present study indicated that M3G, as the typical anthocyanin, performed protection against Cd-induced dysfunction of human granule cells considering the estrogen secretion. Briefly, M3G has a better impact on cell proliferation and cell morphology of CdCl_2_-damaged KGN cells compared to MV and M3,5G. Additionally, M3G treatment in KGN cells that had been damaged by CdCl_2_ decreased ROS production, prevented the arrest of the G2/M phase, and increased estradiol synthesis by alleviating the aberrant expression of the key protein involved in estrogens including CYP17A1 and HSD17B7. As a result, this study provides a basis for M3G as a dietary supplement to protect against Cd-induced damage to female reproduction, which is critical to populations with a high risk of Cd exposure. However, the underlying mechanism by which anthocyanins protect KGN cells from Cd remains unclear.

## Figures and Tables

**Table 1:** Primer sequence for RT-qPCR.

Gene Name	Primer Sequence (5′→3′)	
*Hsd3b*	Forward	CACATGGCCCGCTCCATAC
	Reverse	GTGCCGCCGTTTTTCAGATTC
*Hsd17b1*	Forward	ACGTGAATGTAGTAGGGACTGT
	Reverse	GCGCAATAAACGTCATTGAAAGG
*Hsd17b7*	Forward	ATCTGGACATCATCTCGCAGT
	Reverse	AAGAGCTGTAGGGTTCCTTGC
*Hsd17b12*	Forward	TGTCCCACTCTTGACCATCTAT
	Reverse	CTTGCTCCTATACTCCTCATGGA
*Vps18*	Forward	ACACTGCTCCGCATTGACTT
	Reverse	TTTTGCGTCATCCTTACGTCC
*Vps11*	Forward	CAATCCACTCTGCACTCGAAT
	Reverse	CGGGTGATGTCTCCTTTGTTCA
*Cnot9*	Forward	CACTGGCACAAGTGGATAGAG
	Reverse	GCTTCTTACTTAGCTCCAGCAAA
*Wbp2*	Forward	GCGGAGTGATCGTCAATAACAT
	Reverse	GACCCGGTAAGGGGTAAGGT
*Tiparp*	Forward	AATTTGACCAACTACGAAGGCTG
	Reverse	CAGACTCGGGATACTCTCTCC
*Rara*	Forward	GGGCAAATACACTACGAACAACA
	Reverse	CTCCACAGTCTTAATGATGCACT
*Il4r*	Forward	ACACCAATGTCTCCGACACTC
	Reverse	TGTTGACTGCATAGGTGAGATGA
*Gba*	Forward	GCAGGGCTAACCTAGTGCCT
	Reverse	GCTTGGGACATTCCTCTCTGG
*Kat5*	Forward	AACAAACGTCTGGATGAATGGG
	Reverse	AGGAAGTCCGTTCTTAGTGGG
*Uba5*	Forward	GTTGGTGGAGTAGGTAGTGTGA
	Reverse	GTTCCTCAGAGTATGTTCTGCTG
*Gper1*	Forward	TCACGGGCCACATTGTCAAC
	Reverse	GTCTCCCCGAGAAAGCTGTAG
*Stxbp1*	Forward	AAAGCTGTTGTCGGAGAGAAG
	Reverse	CACAATCGTTATGCCCTCGG
*Gapdh*	Forward	GTCGGAGTCAACGGATTTGG
	Reverse	GGGTGGAATCAATTGGAACAT

Use italics for genes, where the first letter is capitalized and the rest of the letters are lowercase.

## Data Availability

All of the data is contained within the article.
